# Association among Blink Rate, Changes in Ocular Surface Temperature, Tear Film Stability, and Functional Visual Acuity in Patients after Cataract Surgery

**DOI:** 10.1155/2019/8189097

**Published:** 2019-08-19

**Authors:** Takashi Itokawa, Yukinobu Okajima, Takashi Suzuki, Tatsuhiko Kobayashi, Yuto Tei, Koji Kakisu, Yuichi Hori

**Affiliations:** ^1^Department of Ophthalmology, Toho University Graduate School of Medicine, Tokyo, Japan; ^2^Department of Ophthalmology, School of Medicine, Toho University, Tokyo, Japan; ^3^Ishizuchi Eye Clinic, Niihama, Ehime, Japan

## Abstract

**Purpose:**

To investigate the association among the ocular surface temperature (OST), tear film stability, functional visual acuity (FVA), and blink rate in patients after cataract surgery.

**Methods:**

We recruited 98 eyes of 69 patients (mean age, 73.7 ± 5.2 years) 1 month after phacoemulsification with implantation of acrylic intraocular lenses and assessed slit-lamp microscopy, corrected distance VA, FVA, noninvasive tear breakup time (NIBUT), and OST. We defined the changes in the OST from 0 to 10 seconds after eye opening as the ΔOST. We measured the FVA and blink rate using the FVA measurement system. We divided the patients into two groups based on tear film stability: stable tear film (NIBUT, >5.0 seconds) and unstable tear film (NIBUT, ≤5.0 seconds). We evaluated the differences between the two groups and the association between the blink rate and other clinical parameters.

**Results:**

The unstable tear film group (56 eyes) had significantly (*p* < 0.0001, unpaired *t*-test) shorter NIBUTs than the stable tear film group (42 eyes). The ΔOSTs and blink rates were significantly (*p* < 0.0001) higher in the unstable tear film group than in the stable group. Linear single regression analysis showed that the ΔOST (*r* = −0.430, *p* < 0.0001), NIBUT (*r* = −0.392, *p* < 0.0001), and gender (*r* = −0.370, *p*=0.0002) were correlated significantly with the blink rate. Multiple regression analysis showed that the ΔOST independently contributed to the blink rate.

**Conclusions:**

The frequency of blinks is associated with tear film stability in patients after cataract surgery. The blink rate may be useful for evaluating the tear film stability in clinical practice. The ΔOST should be an important contributing factor to the blink rate. [This trial is registered with UMIN000026970].

## 1. Introduction

With improvements in the techniques of cataract surgery, the invasiveness of the surgery has been minimized greatly [1–4]. However, some patients are unsatisfied with the visual quality even though they have good visual acuities (VAs) [5]. Tear film instability on the ocular surface is a possible cause of the problem [6–11]. *After cataract surgery*, *problems with the ocular surface and tear film stability contribute to reduced corneal sensitivity*, *number of goblet cells*, *and mucin expression*, *resulting in decreased tear film breakup time* (*BUT*) [6–10]. These phenomena are believed to impair tear film stability and reduce visual quality. Koh et al. reported that tear film instability degraded the optical quality and increased the higher order aberrations [11].

The ocular surface temperature (OST) represents the physiological function and pathological diagnosis of the ocular surface, and OST has been used to investigate ocular diseases such as dry eye, contact lens discomfort, allergic conjunctivitis, and glaucoma [12–15]. When the tear film is disrupted and becomes unstable, the OST decreases due to evaporation of the tear fluid, convective heat transfer, emission of infrared radiation, and heat conduction [16–21]. Several studies have reported that changes in the OST during eye opening are correlated consecutively with the tear film stability on the cornea [12, 13]. We also reported that changes in the OST are associated with tear film stability not only on the cornea but also over soft contact lenses [13]. Giannaccare et al. reported that 1 month after cataract surgery, the tear film stability decreased and the changes in OST increased compared with preoperative findings. The investigators concluded that the tear film stability and subjective symptoms were correlated with the changes in the OST [22].

Blinking plays an important role in maintaining the normality of the ocular surface. The blink rates of patients with dry eye have been reported to be higher than in normal eyes [23, 24]. Fibers that are sensitive to cold perceive the changes in the OST on the cornea and facilitate the ability to feel dryness and/or coolness on the ocular surface [25–28]. The changes in OST were thought to affect the ocular protective reflex, such as basal tearing and blinking [23–28]. Several researchers have reported that the number of blinks increases with wind stimulation; in contrast, the number of blinks decreases with instillation of artificial tears and topical anesthesia and with wearing of moisture glasses [23, 29]. It should be noted that the number of blinks decreases when the tear film is stable and increases when the tear film is unstable due to the ocular environment and/or external factors. Li and Lin reported that the maximal interblink period was related to the cooling rate of the ocular surface (°C/second) [30].

Functional visual acuity (FVA) measurement has been developed to evaluate the daily visual function [31–33]. The FVA that measures the changes in the VA over time enables detection of impaired visual function that was not detectable based on conventional VA assessment. *Kaido et al*. *reported that the FVA parameters such as the FVA and visual maintenance ratio were correlated significantly with the blink rate*, *which tended to increase with increasing severity of the ocular surface staining scores in patients with dry eye* [34]. Yamaguchi et al. found that the FVA improved significantly after surgery to remove a mildly cataractous lens even though the conventional VA did not change significantly [35]. However, it is unclear if the FVA is associated with the tear film stability after cataract surgery. The aim of the current study was to investigate the relationships among the OST, tear film stability, blink rate, and FVA in patients after cataract surgery.

## 2. Methods

### 2.1. Subjects

We conducted a prospective study at Toho University Omori Medical Center from April 2017 to December 2018. Ninety-eight eyes of 69 patients (44 women, 25 men; average age, 73.7 ± 5.2 years) who underwent cataract surgery were enrolled in this study (Table 1). The inclusion criteria were 60 years of age or older and a corrected distance visual acuity (CDVA) of at least 20/25 or better in patients who had undergone an uneventful phacoemulsification surgery. To avoid variations in the OST, patients who had a history of infection; refractive surgery; corneal diseases such as dry eye, keratoconus, and edema; glaucoma; diabetic retinopathy; allergic conjunctivitis; or a systemic disease such as cancer were excluded.

The FVA, noninvasive tear breakup time (NIBUT), and OST were measured 1 month (average, 31.5 ± 6.0 days) after cataract surgery. The cornea also was assessed for corneal epithelial damage, which was identified by the fluorescein staining scores of the area (*A*) and density (*D*) over the damaged corneal lesions [36]. The degrees of staining of the area and density were scored on a scale from 0 to 3, with A0 indicating none, *A*1 less than one third of the area, *A*2 one third to two thirds, *A*3 more than two thirds, and *D*0 none, *D*1 sparse density, *D*2 moderate density, and *D*3 high density [36]. To investigate the difference in the clinical parameters, i.e., FVA, OST, and corneal staining, based on the effect of tear film stability, the patients were classified into two groups: those with stable tear film (BUT, >5.0 seconds) and unstable tear film (BUT, ≤5.0 seconds). *Although we believe that fluorescein BUT and NIBUT are different*, *no previous reports have statistically investigated the differences between DR-1 interferometry and fluorescein BUT*. In the current study, we set the cutoff line at NIBUT 5.0 seconds according to the 2016 Asia Dry Eye Society criteria, which establish a diagnosis of dry eye with positive subjective symptoms and decreased fluorescein BUT (≤5 seconds). We also measured the blink rate and investigated the association between the blink rate and the other clinical parameters.

The Ethics Committee of Toho University Omori Medical Center approved the study (study protocol, M16246). All patients provided informed consent after they received an explanation of the possible consequences of the study, which adhered to the tenets of the Declaration of Helsinki.

### 2.2. Measurements of Blink Rate and FVA

The temperature (25.2 ± 1.6°C) and humidity (39.2 ± 11.2%) in the examination room were maintained at constant levels. We used the FVA measurement system (Kowa, Aichi, Japan) to measure the FVA and the blink rate. Kaido et al. [31–33] previously described the use of the measurement system. In brief, the FVA was measured monocularly with the best spectacle correction under photopic conditions and natural blinking during a 60-second period. The major outcome parameters were the baseline VA, FVA, visual maintenance ratio (VMR), and blink frequency. The baseline VA was defined as the conventional Landolt CDVA. The FVA was the average value of all VA measurement for 60 seconds. The VMR was defined as the ratio of the FVA value divided by the baseline VA. On the basis of the recorded data for 60 seconds, we defined the total number of blinks as the blink rate (frequency/minute) [31–33].

### 2.3. Measurement of Tear Film Stability

To evaluate the tear film stability, we measured the NIBUT only once to avoid reflex tearing using a tear film interferometer (DR-1*α*, Kowa Co. Ltd., Tokyo, Japan) with low magnification (7.2 × 8.0 mm) [13, 37, 38]. After natural blinking, the patients were asked to keep their eyes open for 10 seconds [13, 38, 39]. The NIBUT was recorded at the first appearance of the breakup of the tear film. When no noninvasive breakup was observed during the 10-second observation period, the NIBUT was recorded as 10 seconds.

### 2.4. Measurement of OST

To measure the OST, we used a noninvasive ocular surface thermographer (TG1000, Tomey, Nagoya, Japan) [12, 13]. The instrument was equipped with a modified optical head of an autorefractor/keratometer (RC-50000, Tomey, Nagoya, Japan) and enabled determination of the central cornea along the optical axis. The method of measuring the OST was the same as described by Mori et al. [40]; i.e, the patients were instructed to close their eyes for 5 seconds and then to keep their eyes open for 10 seconds. The OST was measured in the central cornea (4.0 mm diameter) every second for 10 seconds without blinking. The difference in the OST from 0 to 10 seconds was defined as the ΔOST [12, 13].

### 2.5. Surgical Technique and Postoperative Treatment

Two surgeons (HY and OY) performed all surgeries with the same procedure technique. In all cases, the same methods of lens extraction and intraocular lens implantation were performed with creation of superior corneoscleral incisions (2.4 mm). Postoperatively, 0.1% betamethasone phosphate and 1.5% levofloxacin eye drops (Levaquin, Johnson & Johnson, New Brunswick, NJ, USA) (4 times daily) were used for 1 week, and 0.1% nepafenac ophthalmic solution (Nevanac, Alcon, Ft. Worth, TX, USA) (3 times daily) was used for 4 weeks. Patients were asked to not instill eye drops within 2 hours before the measurements.

### 2.6. Statistical Analysis

The unpaired *t*-test was used to compare the clinical parameters between the stable and unstable tear film groups. Pearson's correlation coefficients and multiple regression analysis were used to identify the independent factors associated with the blink rate. *p* *≦* 0.05 was considered statistically significant. All analyses were conducted using JMP version 11 statistical analysis software (SAS Institute Inc., Cary, NC, USA).

## 3. Results

### 3.1. Comparison of Clinical Parameters between the Stable Tear Film and Unstable Tear Film Groups

**Figure 1:**
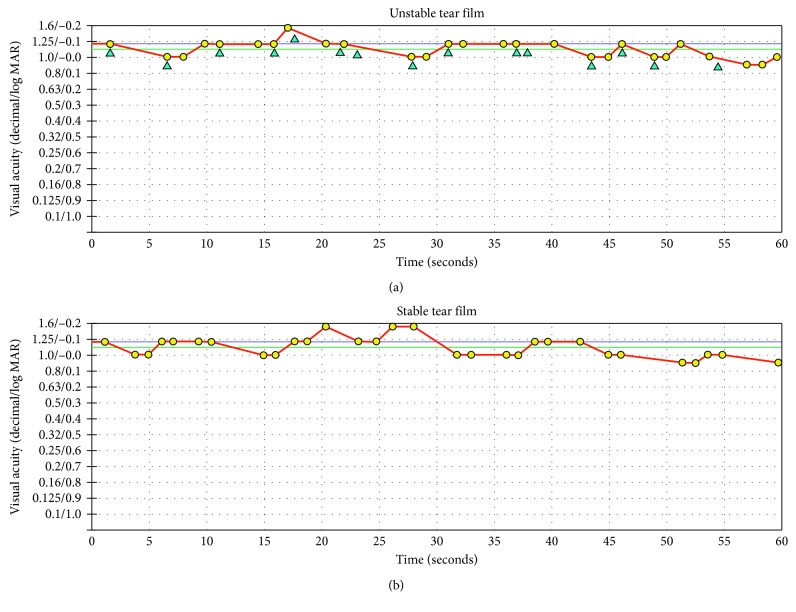
Representative results of functional visual acuity (FVA) in patients after cataract surgery. (a) A 69-year-old woman with unstable tear film (noninvasive tear breakup time (NIBUT), 2.1 seconds; functional visual acuity (FVA) logarithm of minimum angle of resolution (log MAR), −0.05; visual maintenance ratio (VMR), 0.99%; and blink rate (frequency/minute), 15). (b) A 79-year-old man with stable tear film (NIBUT, 10.0 seconds; FVA (log MAR), −0.04; VMR, 0.99%; and blink rate (frequency/minute), 0). The red line indicates the time-wise changes in the visual acuity (VA) for 60 seconds. The blue line indicates the starting VA, and the green line indicates the mean FVA for 60 seconds. The yellow dots indicate the number of correct responses, and the blue triangles indicate the spontaneous blinks.

Of the 98 eyes, 56 eyes were in the stable tear film group, and 42 eyes were in the unstable tear film group. Representative cases of patients with unstable tear film and stable tear film are shown in Figure 1. There was no difference in the FVA, but the blink rate increased in patients with unstable tear film more than in those with stable tear film.  Table 2 shows the comparisons of the clinical parameters between the groups. The NIBUTs in the stable and unstable tear film groups were, respectively, 9.1 ± 1.6 and 3.0 ± 1.1 seconds, a difference that reached significance (*p* < 0.0001, unpaired *t*-test). The respective blink rates in the stable and unstable groups were 4.9 ± 5.8 and 9.3 ± 7.5, a difference that reached significance (*p*=0.0013). The ΔOSTs in the stable and unstable tear film groups were −0.27 ± 0.23°C and −0.56 ± 0.23, respectively, a difference that reached significance (*p* < 0.0001). The NIBUT was correlated significantly with the ΔOST (*r* = 0.607; *p* < 0.0001, Pearson's correlation coefficients). The grading of the corneal epithelial damage, CDVA, FVA, VMR, and OST did not differ significantly between the two groups.

### 3.2. Associations between Blinks and Other Parameters

**Figure 2:**
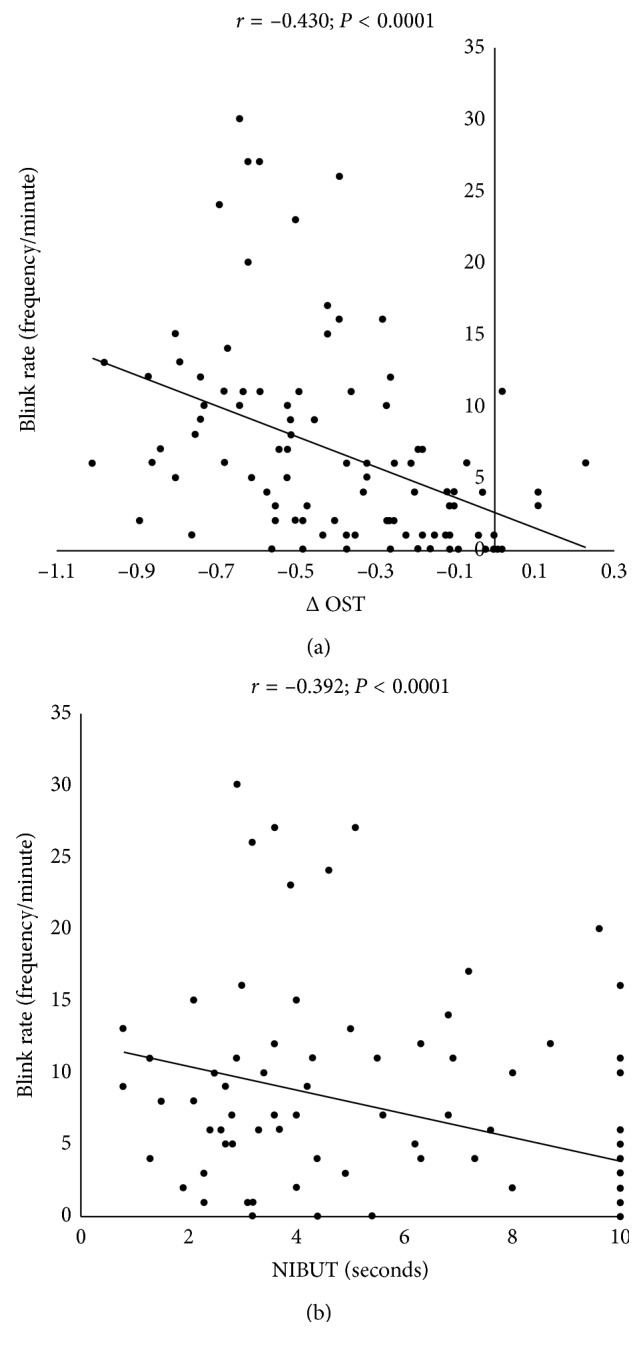
Correlation between the blink rate and the changes in the ocular surface temperature (ΔOST) (a) and noninvasive tear breakup time (NIBUT) (b). ΔOST = difference in the OST from 0 to 10 seconds without blinking.

Table 3 shows the correlation coefficients identified by single regression analysis between the blink rate and other parameters. The blink rate was correlated significantly with the difference in gender (male = 1, female = 0; *r* = −0.370, *p*=0.0002), ΔOST (*r* = 0.430, *p* < 0.0001; Figure 2(a)), and NIBUT (*r* = −0.392, *p* < 0.0001; Figure 2(b)). Age tended to be correlated with the blink rate but did not reach significance (*p*=0.094). From this result, it was found that the blink rate increased when the ΔOST enlarged and the tear film stability became unstable. Regarding gender, the blink rate was higher in women compared to men.

### 3.3. Factors Independently Contributed to the Blink Rate

Table 4 shows the results of multiple regression analysis for factors that independently contributed to the blink rate in the study population. The factor that contributed independently to the blink rate was the ΔOST (*β* = −0.2514; *t*-value = −2.12; *p*=0.0369). When multiple regression analysis was performed with gender, ΔOST, and NIBUT as independent variables, the ΔOST contributed independently to the blink rate. In other words, blinking was triggered by the decrease of the OST following tear film instability.

## 4. Discussion

Some researchers have reported that tear film stability in patients after cataract surgery may change due to decreased corneal sensitivity, reduced number of goblet cells, and mucin expression [6–10]. *Similar to the results of the current study*, *several other studies have proved that the changes in the OST and blink rate increase when the tear film is unstable* [12, 13, 23, 24]. *In addition*, *it was found that the evaluation of the daily visual function*, *measured via the FVA*, *also was related to tear film stability* [34]. However, few studies have attempted to investigate the relationships among the OST, tear film stability, blink rate, and FVA in patients who underwent cataract surgery. In the current study, we found that the blink rate was correlated negatively with the ΔOST and NIBUT. Multiple regression analysis showed that the ΔOST contributed independently to the blink rate.

The OST varied in accordance with the tear film stability. The tear film BUT has been reported to be correlated with the ΔOST [12, 13, 41]. It is well known that tear film evaporation is the major cause of the decreasing OST [16–21]. The current study found that the NIBUT also was correlated with the ΔOST in patients after cataract surgery. This result agreed with a previous study performed by Giannaccare et al. in which both the tear BUT and subjective score were correlated with the ΔOST in patients before and after cataract surgery [22].

Fibers that are sensitive to cold, which perceive the OST, comprise 10% to 15% of the corneal afferents [42]. Cold sensitivity is activated primarily by transient receptor potential M8 (TRPM8), *which responds to temperature reduction and osmotic stimulation*. There are two types of cold thermoreceptors in the cornea: one is activated by a small change in the OST (within 2°C) through which dryness is perceived, and the other is by a large change in the OST through which pain is perceived. Cold thermoreceptors over the cornea play an important role in these sensations [25, 26]. Some researchers have reported that osmotic stimulation and menthol, which are agonists of TRPM8 stimulation, excite the nerves and increase the number of blinks as an ocular protective reflex [43, 44]. In the current study, the ΔOST and blink rate were correlated significantly, i.e., the more the change in the OST increased, the higher the blink rate became.

Blinking plays an important role in maintaining the tear film stability and a healthy ocular surface. Inomata et al. reported that the maximal blink interval was correlated significantly with tear film stability [45]. Rahman et al. found that the blink rate was associated with the degree of ocular surface disease and tear stability [24]. The current results agreed with those previous studies; i.e., the blink rate was correlated with tear film stability in patients after cataract surgery.

Nosch et al. reported that the blink rate and ΔOST were not correlated significantly in young normal subjects [46]. In the current study, however, we recruited elderly people who had undergone cataract surgery. Moreover, their tear film stability distributed to various degree from collected data. We theorized that in cases of tear film instability after cataract surgery, blinking plays a more important protective role in protecting the ocular surface, and the blink rate was correlated significantly with the tear film stability and changes in the OST.

The current results showed that the blink rate was significantly higher in the group with unstable tear film; however, no significant changes were found in the CDVA, corneal staining such as area and density grade, FVA, or VMR between the stable and unstable groups. Kaido et al. reported that the blink rate increased significantly in patients with dry eye even though the FVA and VMR did not change [47]. However, those investigators also reported that the FVA and VMR changed significantly in patients with moderate and severe dry eye with corneal staining but did not change significantly in patients with mild dry eye without corneal staining [34]. Koh et al. also reported that higher order aberrations were higher in patients with dry eye with superficial punctate keratopathy (SPK) on the central cornea than in patients without SPK [11]. In the current study, there were no significant differences in corneal staining grade, FVA, and VMR because we excluded patients with dry eye with severe corneal staining. These data indicate that the FVA could remain at a normal level by increasing the blink rate in cases of abnormal tear film stability; however, in cases in which the corneal epithelial cells are damaged, the FVA may be affected even if the blink rate increases. From the collected data and analysis, we hypothesized that visual abnormalities due to transient tear dysfunction after cataract surgery may be compensated for by increasing the blinking frequency. *Further clinical research is needed to investigate the association among these parameters*, *including higher order aberration in patients with dry eye before and after cataract surgery*.

The current study had several limitations. First, because we enrolled patients who were 60 years of age or older, we could not exclude all systemic diseases such as high blood pressure and diabetes. These systemic diseases might have affected the value of the OST [48]. Second, subjective symptoms were not examined using a questionnaire in this study; the blink rate might have changed depending on the subjective symptoms. *Furthermore*, *we did not evaluate measurements of higher order aberration to investigate visual quality and anterior segment cells and/or flares to evaluate inflammation*; *these data may explain the degraded visual quality and relationship between OST and inflammation*. Finally, in this study, the number of blinks was counted during measurement of the FVA, which means that the blink rate could not be measured under natural conditions.

## 5. Conclusions

*Although FVA*, *VMR*, *and corneal epithelial damage grade did not show significant differences between the groups with stable and unstable tear film*, *the blink rate and* Δ*OST showed significant differences*. The blink rate was correlated significantly with the ΔOST and tear film stability in patients after cataract surgery, and the ΔOST contributed independently to the blink rate. The FVA in patients with unstable tear film after cataract surgery might remain in the normal range by increasing the blink rate.

## Figures and Tables

**Table 1:** Demographic data.

	Stable tear film (56 eyes)		Unstable tear film (42 eyes)	
Mean patient age (years)	74.7 ± 5.3 (range, 74.2–76.0)		72.5 ± 4.7 (range, 70.9–74.0)	
	Women (29 eyes)	Men (27 eyes)	Women (36 eyes)	Men (6 eyes)
Age by gender (years)	75.0 ± 5.0 (range, 73.0–76.9)	74.3 ± 5.7 (range, 72.1–76.6)	72.3 ± 4.9 (range, 70.6–73.9)	73.8 ± 3.7 (range, 70.0–77.7)

The data are expressed as the average ± deviation (95% confidence interval).

**Table 2:** Comparison of clinical parameters between the groups.

	Stable tear film (56 eyes)	Unstable tear film (42 eyes)	*p* value
Area	0.2 ± 0.5 (0.0–0.4)	0.3 ± 0.6 (0.1–0.5)	0.3529
Density	0.3 ± 0.7 (0.1–0.4)	0.3 ± 0.7 (0.1–0.6)	0.5560
NIBUT (seconds)	9.1 ± 1.6 (8.7–9.4)	3.0 ± 1.1 (2.6–3.5)	<0.0001
CDVA (log MAR)	−0.04 ± 0.06 (−0.06 to–0.03)	−0.02 ± 0.07 (−0.04 to–0.01)	0.1052
FVA (log MAR)	0.10 ± 0.12 (0.07 to 0.13)	0.11 ± 0.13 (0.07 to 0.14)	0.8179
VMR (%)	0.95 ± 0.04 (0.94 to 0.96)	0.95 ± 0.03 (0.94 to 0.96)	0.7175
Blink rate (frequency/minute)	4.9 ± 5.8 (3.1 to 6.6)	9.3 ± 7.5 (7.3 to 11.3)	0.0013
OST (°C)	34.41 ± 0.57 (34.26 to 34.56)	34.43 ± 0.57 (34.25 to 34.56)	0.8310
ΔOST (°C)	−0.27 ± 0.23 (−0.32 to–0.20)	−0.56 ± 0.23 (−0.63 to–0.49)	<0.0001

The data are expressed as the average ± deviation (95% confidence interval). Compared with the unstable tear film group, the NIBUT, ΔOST and blink rate differ significantly in the stable tear film group. log MAR: logarithm of the minimum angle of resolution; NIBUT: noninvasive tear breakup time; CDVA: corrected distance visual acuity; FVA: functional visual acuity; OST: ocular surface temperature; VMR: visual maintenance ratio; ΔOST: difference in OST from 0 to 10 seconds without blinking.

**Table 3:** Correlations between the blink rate and other parameters.

Explanatory variables	*r* value	*p* value
Men = 1; women = 0	−0.370	0.0002
Age	−0.170	0.0940
NIBUT	−0.392	<0.0001
ΔOST	−0.430	<0.0001
OST	0.004	0.9680
FVA	−0.001	0.9938
VMR	0.067	0.5126

NIBUT: noninvasive tear breakup time; FVA: functional visual acuity; VMR: visual maintenance ratio; OST: ocular surface temperature; ΔOST: difference in OST from 0 to 10 seconds without blinking.

**Table 4:** Results of multiple regression analysis for factors independently contributing to the blink rate.

Variable		*β*	*p* value
Dependent	Independent		
Blink rate	Men = 1; women = 0	−0.1693	0.1211
	NIBUT	−0.1564	0.1888
	ΔOST	−0.2514	0.0369

NIBUT: noninvasive tear breakup time; ΔOST: difference in OST from 0 to 10 seconds without blinking.

## Data Availability

The data used to support the findings of this study are available from the first author, Tadashi Itokawa (takashi.itokawa@med.toho-u.ac.jp).

## References

[1] Stepherd J. R. (1989). Induced astigmatism in small incision cataract surgery. *Journal of Cataract & Refractive Surgery*.

[2] Martin R. G., Sanders D. R., Miller J. D., Cox C. C., Ballew C. (1993). Effect of cataract wound incision size on acute changes in corneal topography. *Journal of Cataract & Refractive Surgery*.

[3] Agarwal A., Agarwal A., Agarwal S., Narang P., Narang S. (2001). Phakonit: phacoemulsification through 0.9 mm corneal incision. *Journal of Cataract & Refractive Surgery*.

[4] Tsuneoka H., Shiba T., Takahashi Y. (2001). Feasibility of ultrasound cataract surgery with a 1.4 mm incision. *Journal of Cataract & Refractive Surgery*.

[5] Marcos S., Rosales P., Llorente L., Jimenez-Alfaro I. (2007). Change in corneal aberrations after cataract surgery with 2 types of aspherical intraocular lenses. *Journal of Cataract & Refractive Surgery*.

[6] Oh T., Jung Y., Chang D., Kim J., Kim H. (2012). Changes in the tear film and ocular surface after cataract surgery. *Japanese Journal of Ophthalmology*.

[7] Li X.-M., Hu L., Hu J., Wang W. (2007). Investigation of dry eye disease and analysis of the pathogenic factors in patients after cataract surgery. *Cornea*.

[8] Han K. E., Toon S. C., Ahn J. M. (2014). Evaluation of dry eye and meibomian gland dysfunction after cataract surgery. *American Journal of Ophthalmology*.

[9] Xue W., Zhu M. M., Zhu B. J. (2019). Long-term impact of dry eye symptoms on vision-related quality of life after phacoemulsification surgery. *International Ophthalmology*.

[10] Cetinkaya S., Mestan E., Acir N. O., Cetinkaya Y. F., Dadaci Z., Yener H. I. (2015). The course of dry eye after phacoemulsification surgery. *BMC Ophthalmology*.

[11] Koh S., Maeda N., Hirohara Y. (2008). Serial measurements of higher-order aberrations after blinking in patients with dry eye. *Investigative Opthalmology & Visual Science*.

[12] Kamao T., Yamaguchi M., Kawasaki S., Mizoue S., Shiraishi A., Ohashi Y. (2011). Screening for dry eye with newly developed ocular surface thermographer. *American Journal of Ophthalmology*.

[13] Itokawa T., Okajima Y., Suzuki T. (2018). Association between ocular surface temperature and tear film stability in soft contact lens wearers. *Investigative Opthalmology & Visual Science*.

[14] Hara Y., Shiraishi A., Yamaguchi M., Kawasaki S., Uno T., Ohashi Y. (2014). Evaluation of allergic conjunctivitis by thermography. *Ophthalmic Research*.

[15] Kawasaki S., Mizoue S., Yamaguchi M. (2009). Evaluation of filtering bleb function by thermography. *British Journal of Ophthalmology*.

[16] Scott J. A. (1988). A finite element model of heat transport in the human eye. *Physics in Medicine and Biology*.

[17] Mapstone R. (1968). Determinants of corneal temperature. *British Journal of Ophthalmology*.

[18] Lagenkijk J. J. (1982). A mathematical model to calculate temperature distributions in human and rabbit eyes during hypothermic treatment. *Physics in Medicine and Biology*.

[19] Tan J. H., Ng E. Y., Acharya U. R. (2010). Evaluation of tear evaporation from ocular surface by functional infrared thermography. *Medical Physics*.

[20] Li W., Graham A. D., Selvin S., Lin M. C. (2015). Ocular surface cooling corresponds to tear film thinning and breakup. *Optometry and Vision Science*.

[21] Su T. Y., Chang S. W., Yang C. J., Chiang H. K. (2014). Direct observation and validation of fluorescein tear film break-up patterns by using a dual thermal-fluorescent imaging system. *Biomedical Optics Express*.

[22] Giannaccare G., Fresina M., Agnifili L., Versura P. (2016). Ocular-surface temperature modification by cataract surgery. *Journal of Cataract & Refractive Surgery*.

[23] Nakamori K., Odawara M., Nakajima T., Mizutani T., Tsubota K. (1997). Blinking is controlled primarily by ocular surface conditions. *American Journal of Ophthalmology*.

[24] Rahman E. Z., Lam P. K., Chu C. K., Moore Q., Pflugfelder S. C. (2015). Corneal sensitivity in tear dysfunction and its correlation with clinical parameters and blink rate. *American Journal of Ophthalmology*.

[25] Belmonte C., Gallar J. (2011). Cold thermoreceptors, unexpected players in tear production and ocular dryness sensations. *Investigative Opthalmology & Visual Science*.

[26] Belmonte C., Acosta M. C., Merayo-Lioves J., Gallar J. (2015). What causes eye pain?. *Current Ophthalmology Reports*.

[27] Hirata H., Mizerska K., Dallacasagrande V., Rosenblatt M. I. (2017). Estimating the osmolarities of tears during evaporation through the “eyes” of the corneal nerves. *Investigative Opthalmology & Visual Science*.

[28] Hirata H., Dallacasagrande V., Mizerska K., Ivakhnitskaia E., Rosenblatt M. I. (2018). Ambient air currents activate corneal nerves during ocular desiccation in rats: simultaneous recordings of neural activity and corneal temperature. *Investigative Opthalmology & Visual Science*.

[29] Ogawa M., Dogru M., Toriyama N., Yamaguchi T., Shimazaki J., Tsubota K. (2018). Evaluation of the effect of moist chamber spectacles in patients with dry eye exposed to adverse environment conditions. *Eye & Contact Lens*.

[30] Li W., Lin M. C. (2018). Pain sensitivity associated with the length of the maximum interblink period. *Investigative Opthalmology & Visual Science*.

[31] Goto E., Yagi Y., Matsumoto Y., Tsubota K. (2002). Impaired functional visual acuity of dry eye patients. *American Journal of Ophthalmology*.

[32] Kaido M. (2018). Functional visual acuity. *Investigative Opthalmology & Visual Science*.

[33] Kaido M., Dogru M., Ishida R., Tsubota K. (2007). Concept of functional visual acuity and its applications. *Cornea*.

[34] Kaido M., Ishida R., Dogru M., Tsubota K. (2011). The relation of functional visual acuity measurement methodology to tear functions and ocular surface status. *Japanese Journal of Ophthalmology*.

[35] Yamaguchi T., Negishi K., Dogru M., Saiki M., Tsubota K. (2009). Improvement of functional visual acuity after cataract surgery in patients with good pre-and postoperative spectacle-corrected visual acuity. *Journal of Refractive Surgery*.

[36] Miyake K., Amano S., Sawa M., Nishida T. (2003). A novel grading method for superficial punctate keratopathy magnitude and its correlation with corneal epithelial permeability. *Archives of Ophthalmology*.

[37] Ishibashi T., Yokoi N., Kinoshita S. (2003). Comparison of the short-term effects on the human corneal surface of topical timolol maleate with and without benzalkonium chloride. *Journal of Glaucoma*.

[38] Yokoi N., Georgiev G. A., Kato H. (2017). Classification of fluorescein breakup patterns: a novel method of differential diagnosis for dry eye. *American Journal of Ophthalmology*.

[39] Maruyama K., Yokoi N., Takamata A., Kinoshita S. (2004). Effect of environmental conditions on tear dynamics in soft contact lens wearers. *Investigative Opthalmology & Visual Science*.

[40] Mori A., Oguchi Y., Okusawa Y., Ono M., Fujishima H., Tsubota K. (1997). Use of high-speed, high-resolution thermography to evaluate the tear film layer. *American Journal of Ophthalmology*.

[41] Morgan P. B., Tullo A. B., Efron M. (1996). Ocular surface cooling in dry eye-a pilot study. *Journal of the British Contact Lens Association*.

[42] Belmonte C., Aracil A., Acosta M. C., Luna C., Gallar J. (2004). Nerves and sensations from the eye surface. *The Ocular Surface*.

[43] Quallo T., Vastani N., Horridge E. (2015). TRPM8 is a neuronal osmosensor that regulates eye blinking in mice. *Nature Communications*.

[44] Kovacs I., Luna C., Quirce S. (2016). Abnormal activity of corneal cold thermoreceptors underlies the unpleasant sensations in dry eye disease. *Pain*.

[45] Inomata T., Iwagami M., Hiratsuka Y. (2018). Maximum blink interval is associated with tear film breakup time: a new simple, screening test for dry eye disease. *Scientific Reports*.

[46] Nosch D. S., Pult H., Albon J., Purslow C., Murphy P. J. (2016). Relationship between corneal sensation, blinking, and tear film quality. *Optometry and Vision Science*.

[47] Kaido M., Kawashima M., Shigeno Y., Yamada Y., Tsubota K. (2017). Relation of accommodative microfluctuation with dry eye symptoms in short tear break-up time dry eye. *PLoS One*.

[48] Morgan P. B., Smyth J. V., Tullo A. B., Efron N. (1999). Ocular temperature in carotid artery stenosis. *Optometry and Vision Science*.

